# Characterisation of functional domains in fission yeast Ams2 that are required for core histone gene transcription

**DOI:** 10.1038/srep38111

**Published:** 2016-11-30

**Authors:** Yuko Takayama, Masaki Shirai, Fumie Masuda

**Affiliations:** 1 Department of Biosciences, School of Science and Engineering, Teikyo University, Utsunomiya, Tochigi, 320-8551, Japan; 2 Division of Integrated Science and Engineering, Teikyo University Graduate School of Science and Engineering, Utsunomiya, Tochigi, 320-8551, Japan; 3 Division of Cell Biology, Institute of Life Science, Kurume University, Kurume, Fukuoka, 839-0864, Japan

## Abstract

Histone gene expression is regulated in a cell cycle-dependent manner, with a peak at S phase, which is crucial for cell division and genome integrity. However, the detailed mechanisms by which expression of histone genes are tightly regulated remain largely unknown. Fission yeast Ams2, a GATA-type zinc finger motif-containing factor, is required for activation of S phase-specific core histone gene transcription. Here we report the molecular characterisation of Ams2. We show that the zinc finger motif in Ams2 is necessary to bind the histone gene promoter region and to activate histone gene transcription. An N-terminal region of Ams2 acts as a self-interaction domain. Intriguingly, N-terminally truncated Ams2 binds to the histone gene promoters, but does not fully activate histone gene transcription. These observations imply that Ams2 self-interactions are required for efficient core histone gene transcription. Moreover, we show that Ams2 interacts with Teb1, which itself binds to the core histone gene promoters. We discuss the relationships between Ams2 domains and efficient transcription of the core histone genes in fission yeast.

In eukaryotic cells, the nucleosome is the basic unit of chromatin. The nucleosome is an octamer comprising two of each of the four core histone proteins (H2A, H2B, H3, and H4). Appropriate transcription levels of core histone genes are essential for several chromatin-mediated processes, chromosome segregation, and cell viability^1^,^2^,^3^. The core histone genes show evolutionarily conserved organisation^4^. In higher eukaryotes, core histone genes are highly iterated and organised into clusters^4^,^5^, whereas the budding yeast *Saccharomyces cerevisiae* harbours two copies of each pair of the genes encoding H2A-H2B and H3-H4^6^,^7^,^8^. In the fission yeast *Schizosaccharomyces pombe*, the genomic organisation consists of a single H2Aβ-encoding gene (*hta2*^+^), a single pair of H2Aα-H2B -encoding genes (*hta1*^+^*-htb1*^+^), and three pairs of H3-H4 -encoding genes (*hht1*^+^*-hhf1*^+^, *hht2*^+^*-hhf2*^+^, *hht3*^+^*-hhf3*^+^)^9^,^10^. Histone gene transcription is increased at S phase, coinciding with nucleosome assembly during DNA synthesis. In metazoans, accumulation of histone mRNA employs both transcriptional and post-transcriptional mechanisms^11^. In contrast, the accumulation of core histone gene transcripts in fission yeast is regulated primarily at the level of transcriptional activation, and is strictly repressed outside of the S phase^12^.

Previous studies showed that ectopic expression of core histone genes outside of S phase leads to chromosome instability and cell toxicity^3^. Various factors are known to be required for S phase-specific transcriptional activation. In budding yeast, the Spt10 and Spt21 proteins physically interact with the histone gene promoter and cooperatively activate histone gene transcription^13^,^14^. Spt21 protein accumulates at S phase and binds to the histone gene promoters in a Spt10-dependent manner. Spt10 contains a C2H2-type zinc finger motif that binds to upstream activation sequence (UAS) elements in the histone gene promoters^15^. A protein fragment comprising Spt10’s DNA-binding domain (DBD; the zinc finger motif) alone, binds with high affinity to a single UAS region. However, when Spt10-DBD is fused with the Spt10 N-terminal domain (NTD), the fused protein is not able to bind a single UAS element, although the combination is able to bind a target that includes two UAS elements. Notably, paired UAS elements are found exclusively in the promoters of the core histone genes. Based on these observations, it has been proposed that Spt10 proteins containing NTD and DBD form dimers via an N-terminal domain that stably binds to paired UAS elements^7^,^16^.

In fission yeast, Ams2, a GATA-type zinc finger protein, promotes the centromere localisation of the centromere-specific histone H3 variant CENP-A^17^. Ams2 is required for transcriptional activation of the core histone genes at S phase, and binds to the histone gene promoter regions *in vivo* in an AACCCT-box dependent manner^12^. The AACCCT-box is a 17-bp DNA sequence that is present in all core histone gene promoters^9^,^10^. Ams2 protein levels, which are controlled by the ubiquitin proteasome pathways^18^,^19^, increase during the cell cycle to a peak in the G1/S phase^12^,^17^ that ensures S phase-specific transcriptional activation of core histone genes. A recent study reported that phosphorylation of histone H3 serines 86 and 87 is another mechanism for controlling Ams2 protein expression^20^. However, although Ams2 cell cycle-dependent protein stabilisation is an important aspect of Ams2 function, the molecular and functional characteristics of Ams2 domains remain unknown. In this study, we examined the roles of the zinc finger motif and N-terminal domains of Ams2 in core histone gene transcription.

## Results

### The zinc finger motif of Ams2 is necessary to bind the core histone gene promoters

**Figure 1:**
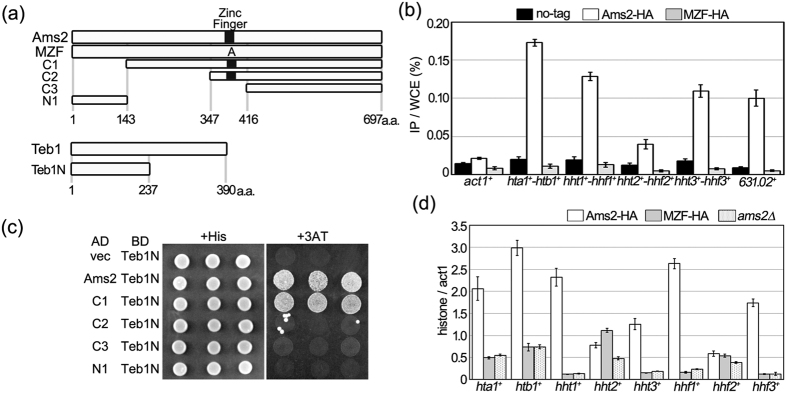
The zinc finger motif of Ams2 is necessary for binding the core histone gene promoters. (**a**) Schematic representation of Ams2 or Teb1 deletion mutants. Black boxes show zinc finger motifs. Amino acid residue numbering is indicated below each panel. (**b**) Cell extracts for use in ChIP assay were prepared from cells expressing untagged wild-type Ams2 (HM123), HA-tagged Ams2 (YTP894), or HA-tagged zinc finger-mutated Ams2 (MZF, YTP1387). Cells were grown in YES medium at 33 °C and arrested in S phase by exposure to 12 mM HU for 3.5 h at 33 °C. DNAs co-immunoprecipitated with anti-HA antibody were quantified by real-time PCR using promoter region probes specific for *act1*^+^, individual histone genes, or the *SPAC631.02*^+^ gene. The amount of each immunoprecipitated DNA was divided by that of the corresponding whole-cell extract DNA after background titration. The error bars indicate the standard deviation from two independent immunoprecipitation assays and duplicate PCR experiments. (**c**) Various fragments from Ams2 were cloned into the pGAD (AD) plasmid. Each plasmid was transformed into the L40 strain along with pBTM (BD)-Teb1N. The resulting transformants were grown at 30 °C on medium supplemented with histidine (+His), or on medium lacking histidine and supplemented with 25 mM 3-AT (+3AT). (**d**) Total RNA was prepared from cells encoding HA-tagged Ams2 (YTP894) or HA-tagged zinc finger-mutated Ams2 (MZF, YTP1387), or from cells lacking *ams2*^+^ (*ams2Δ*, YTP112). Strains were grown in YES medium at 33 °C and arrested in S phase by exposure to 12 mM HU for 3.5 h at 33 °C. The transcriptional levels of the histone genes were assessed by quantitative RT-PCR. The relative levels of each transcript were calculated by normalising to that of *act1*^+^ transcript. The error bars indicate the standard deviation from two independent cultures.

Ams2 is required for activation of the transcription of the S phase-specific core histone genes^12^. It is widely known that the zinc finger is a DNA-binding motif. To determine whether the zinc finger motif of Ams2 is required for histone gene promoter binding, a chromatin immunoprecipitation (ChIP) assay was performed. We used site-directed mutagenesis to generate a mutated version of *ams2*^+^ encoding a protein harbouring alanine substitutions in two of four cysteine residues (C375A and C378A) of the zinc finger (Fig. 1a). We then constructed strains in which genes encoding HA-tagged versions of Ams2 with or without the mutated zinc finger (MZF) were separately integrated at the chromosomal *ams2*^+^ locus, permitting expression of the tagged proteins from the native promoter. These cells were synchronised in S phase by HU treatment, and then the cell lysates were immunoprecipitated with anti-HA antibody. Wild-type Ams2-HA accumulated on core histone gene promoters (Fig. 1b, white bars), as previously reported^12^. In contrast, the accumulation of zinc finger-mutated Ams2-HA on core histone gene promoters was greatly compromised (Fig. 1b, grey bars). Ams2 protein also binds to the upstream region of non-histone gene (*SPAC631.02*^+^), which contains the AACCCT-box^12^. We further examined whether zinc finger-mutated Ams2 associates with the *SPAC631.02*^+^ gene promoter region. Notably, we did not detect association of the zinc finger-mutated Ams2 with the *SPAC631.02*^+^ gene promoter (Fig. 1b, *631.02*^+^). These results clearly showed that the zinc finger motif of Ams2 is required for binding to the AACCT-box, which is contained in all core histone gene promoters.

It has been reported that histone gene promoter binding by Ams2 depends on the presence of Teb1, a protein that binds the TTAGGG motif in the AACCCT-box^21^. To elucidate the region of Ams2 that permits binding to Teb1, a yeast two-hybrid assay was performed. We observed that constructs encoding fusions of a DNA-binding domain (BD) with full-length or C-terminal fragment Teb1 resulted in false-positive expression. Instead, we used a construct that encoded the BD fused with an N-terminal fragment of Teb1 (Teb1N; corresponding to amino acid residues 1–237, Fig. 1a), along with constructs that encoded either full-length or N-terminus-deleted Ams2 protein fused with the activation domain (AD). As shown in Fig. 1c, cells harbouring BD-Teb1N and AD-full-length Ams2 were able to grow on 3-amino-1,2,4,-triazole plates (+3AT), indicating physical association between the Teb1 and Ams2 proteins. Moreover, Teb1N was able to associate with the “C1” C-terminal fragment of Ams2 (corresponding to amino acid residues 143–697), but no longer interacted with the “C2” C-terminal fragment of Ams2 (corresponding to amino acid residues 347–697). These results indicated that the N-terminal region of Teb1 binds to an internal domain of Ams2 (amino acids 143–347), a domain that notably does not contain the zinc finger motif.

We assessed transcription levels of the core histone genes in cells encoding the Ams2 zinc finger-mutated protein. Cells encoding chromosomal HA-tagged wild-type or zinc finger-mutated Ams2 were synchronised in S phase by HU treatment and used for RNA preparation, and transcript levels were determined by quantitative RT-PCR. In cells encoding HA-tagged Ams2, core histone gene transcripts were readily detected (Fig. 1d). In contrast, expression levels of core histone gene transcripts were decreased in zinc finger-mutated Ams2 (MZF-HA) -encoding cells. Quantification showed that histone gene transcription levels in the MZF-HA-encoding cells were as low as those in *ams2Δ* cells, in which S phase core histone transcription is inactivated^12^. The sole exception was *hht2*^+^, for which transcript levels were increased (Fig. 1d). We previously reported that, in the absence of other copies of *hht1*^+^, *hhf1*^+^, *hht3*^+^, and *hhf3*^+^, the transcription levels of *hht2*^+^ and *hhf2*^+^ are increased in asynchronous cells^12^. These results indicated that the zinc finger motif of Ams2 is necessary to bind the histone gene promoter region, which is a prerequisite for activation of histone gene transcription by Ams2 associated with Teb1.

### Overproduction of the zinc finger-mutated protein is toxic in CENP-A mutant cells

**Figure 2:**
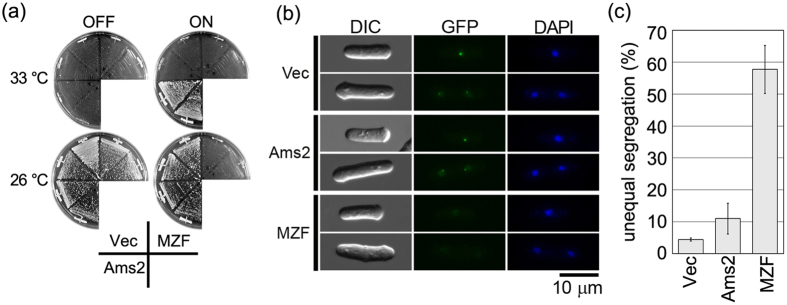
Overproduced zinc finger-mutated Ams2 protein is toxic to *cnp1-1* cells. (**a**) *cnp1-1* (Sp525) cells containing pRep41 (Vec), pRep41-Ams2 (Ams2), or pRep41-zinc finger-mutated Ams2 (MZF) were grown on minimal medium in the absence (ON) or presence (OFF) of thiamine for 4 days at 33 °C or for 6 days at 26 °C. (**b**) Cnp1^ts^-GFP -expressing cells (Sp1102) containing pRep41 (vec), pRep41-Ams2 (Ams2), or pRep41-zinc finger-mutated Ams2 (MZF) were grown on minimal medium in the absence (ON) of thiamine for 30 h at 26 °C. The cells were fixed with methanol at –80 °C and washed with phosphate-buffered saline (PBS). Each of the images shown was captured using the same exposure time. (**c**) *cnp1-1* (Sp525) cells containing pRep41 (Vec), pRep41-Ams2 (Ams2), or pRep41-zinc finger-mutated Ams2 (MZF) were grown in minimal medium in the absence of thiamine at 26 °C for 30 h. The frequency of unequal segregation was measured in binucleate cells from two independent transformants and duplicate experiments (n = 100/strain).

Ams2 was originally identified as one of the four multicopy suppressors of *cnp1-1*, which is a temperature-sensitive (ts) allele of the gene encoding the CENP-A protein (Cnp1 in fission yeast)^17^. We previously hypothesised that accumulation of canonical histone proteins induced by the overproduction of Ams2 enhanced the formation of a Cnp1^ts^-containing nucleosome and its centromere localisation^22^. If this notion is correct, the overexpression of zinc finger-mutated Ams2, which does not activate histone gene transcription (Fig. 1d), should not suppress the *cnp1-1* ts phenotype. To test this possibility, plasmids encoding wild-type Ams2 or zinc finger-mutated Ams2 under the control of the *nmt1-41* promoter (pRep41-Ams2 or pRep41-MZF, respectively) were introduced into *cnp1-1* cells. At the restrictive temperature on a minimal medium plate without thiamine (derepressed condition), wild-type Ams2-overproducing *cnp1-1* cells formed colonies, as previously shown, whereas zinc finger-mutated Ams2-overproducing *cnp1-1* cells showed impaired growth (Fig. 2a, ON, at 33 °C). These results supported the hypothesis that the zinc finger motif of Ams2 is required for the activation of core histone gene transcription. Surprisingly, at the permissive temperature, zinc finger-mutated Ams2-overproducing *cnp1-1* cells still showed severe growth defects (Fig. 2a, ON, at 26 °C); this observation was in contrast to the growth of wild-type Ams2-overproducing *cnp1-1* cells. To confirm whether the Cnp1^ts^ protein is localised to the centromere, we used a strain in which a version of *cnp1-1* encoding GFP-tagged Cnp-1^ts^ was integrated at the *lys1*^+^ locus in an otherwise wild-type background^22^. Consistent with a previous report^17^, overexpression of wild-type Ams2 in this strain resulted in enhanced centromere-localised GFP signal (Fig. 2b, Ams2). In contrast, overexpression of zinc finger-mutated Ams2 in this strain yielded diminished centromere-localised GFP signal (Fig. 2b, MZF). Since centromere localisation of Cnp1 is essential for accurate chromosome segregation^23^, we analysed the profile of chromosome segregation in cells overproducing zinc finger-mutated Ams2. As shown in Fig. 2c and Supplementary Fig. S1, cells overproducing zinc finger-mutated Ams2, but not those overproducing wild-type Ams2, showed a >5-fold-increased frequency of unequal chromosome segregation at the permissive temperature compared to the vector control. These observations indicated that overproduced zinc finger-mutated Ams2 induces chromosome missegregation and growth defects in *cnp1-1* cells, even under permissive conditions.

### Overproduction of zinc finger-mutated Ams2 inhibits histone gene promoter binding by Ams2 expressed from the chromosomal locus

**Figure 3:**
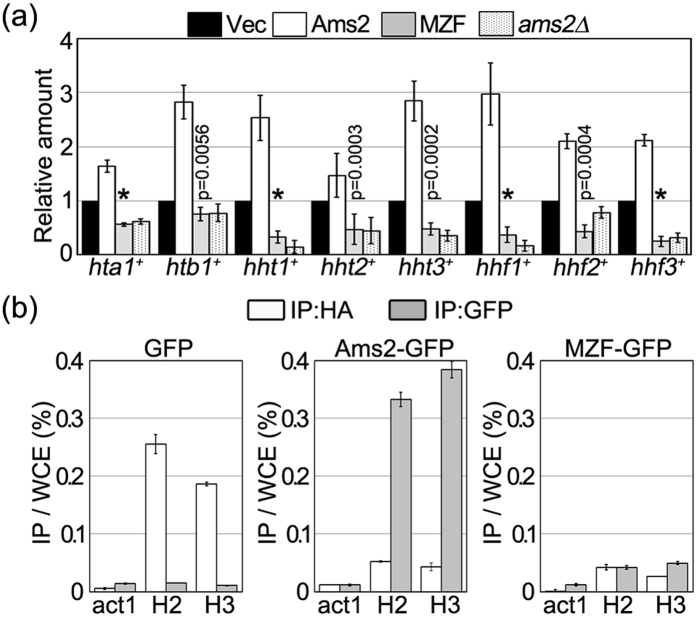
Overproduced zinc finger-mutated Ams2 protein inhibits histone gene promoter binding by Ams2 expressed from the chromosomal locus. (**a**) Wild-type (HM123) cells containing pRep41 (Vec, black bars), pRep41-Ams2 (Ams2, white bars), or pRep41-zinc finger mutant (MZF, grey bars), or cells lacking *ams2*^+^ (*ams2Δ*, YTP112, dotted bars) were grown in minimal medium in the absence (ON) of thiamine at 33 °C for 22 h. The transcript levels were calculated from two independent cultures. The relative amounts of each transcript were divided by those detected in the corresponding vector control after normalising to that of *act1*^+^ mRNA. p values for comparison with vector control were calculated using a Student’s t-test. Asterisks indicate p < 0.0001. (**b**) Cell lysates were prepared from cells expressing HA-tagged Ams2 (YTP894) containing pRep41-GFP (GFP, left panel), pRep41-Ams2-GFP (Ams2-GFP, middle panel), or pRep41-zinc finger-mutated Ams2-GFP (MZF-GFP, right panel). Cells were grown in minimal medium in the absence (ON) of thiamine at 33 °C for 18 h and arrested in S phase by exposure to 12 mM HU for 3.5 h. DNAs co-immunoprecipitated with anti-HA or anti-GFP antibodies were quantified by real-time PCR using promoter region probes specific for the promoters for *act1*^+^ (act1), *hta1*^+^ -*htb1*^+^(H2), or *hht1*^+^-*hhf1*^+^(H3). The amount of each immunoprecipitated DNA was divided by that of the corresponding whole-cell extract DNA after background titration. The error bars indicate the standard deviation from two independent cultures.

The next question was why overproduced zinc finger-mutated Ams2 induces chromosome missegregation and growth inhibition in *cnp1-1* cells. A previous study showed that *ams2*^+^ null (*ams2Δ*) cells exhibit a decrease in the number of CENP-A nucleosomes at the centromere and increased chromosome missegregation^17^. Despite the presence of a wild-type *ams2*^+^ gene in *cnp1-1* cells, zinc finger-mutated Ams2-overproducing *cnp1-1* cells displayed a high rate of chromosome missegregation (Fig. 2c, MZF). Also, the *cnp1-1 ams2Δ* double mutant did not form colonies at the permissive temperature (Supplementary Fig. S2). In *ams2Δ* cells, the total levels of the core histone gene transcripts are decreased compared with those in wild-type cells^12^. Thus, the synthetic lethality between *cnp1-1* and *ams2Δ* suggested that *cnp1-1* cells cannot tolerate decreased expression of core histones. Our interpretation of this result was that overproduction of zinc finger-mutated Ams2 leads to impaired histone gene transcription. To investigate this interpretation, we tested whether the levels of core histone gene transcripts were decreased by overproduction of zinc finger-mutated Ams2 (MZF), even in the presence of the *ams2*^+^ gene. Wild-type cells were transformed with pRep41-Ams2 or pRep41-zinc finger-mutated Ams2 plasmids, and mRNAs were prepared from the resulting cells for quantitative RT-PCR. In Ams2-overproducing wild-type cells, increased levels of histone gene transcripts were detected compared with empty vector controls (Fig. 3a, white bars). In contrast, in zinc finger-mutated Ams2-overproducing cells, the core histone gene mRNA levels were decreased to levels similar to those observed in *ams2Δ* cells (Fig. 3a, grey and dotted bars). Moreover, we confirmed that when the zinc finger-mutated protein was overproduced in *cnp1-1* cells, the histone gene transcript levels also appeared to be decreased (Supplementary Fig. S3b). These findings indicated that core histone gene transcription is repressed in a dominant negative fashion by overproduced zinc finger-mutated Ams2 protein. Therefore, the chromosome missegregation and growth defect in cells overproducing zinc finger-mutated Ams2 are significantly influenced by the decrease in histone gene transcription.

Next, we investigated whether the overproduced zinc finger-mutated Ams2 protein affects the binding to the histone gene promoters of Ams2 produced from the chromosomal locus. To distinguish between Ams2 from the chromosomal locus and the overproduced wild-type or zinc finger-mutated Ams2 proteins, the chromosomal *ams2*^+^ gene was altered to encode wild-type HA-tagged protein, and the overproduced genes under the control of the *nmt1-41* promoter on the plasmid were altered to encode GFP-tagged wild-type or zinc finger-mutated Ams2 proteins. Fission yeast cells in asynchronous culture are mainly in G2 phase. Since the Ams2-HA protein from the chromosomal locus is expressed and accumulates only at S phase, Ams2-HA is barely detectable in asynchronous culture cells. In order to assess DNA binding by Ams2 proteins expressed from the chromosomal and plasmid genes, the resulting cells were arrested in S phase, and ChIP assays were performed using anti-HA or anti-GFP antibodies. In the control strain, which overproduced GFP alone, the Ams2-HA protein from the chromosomal locus accumulated at core histone gene promoters (*hta1*^+^ - *htb1*^+^ (H2), *hht1*^+^ - *hhf1*^+^ (H3) in Fig. 3b, left panel). In the strain that overproduced GFP-tagged wild-type Ams2, the Ams2-GFP bound preferentially to the histone gene promoters, whereas the accumulation of Ams2-HA from the chromosomal locus at these promoters was decreased (Fig. 3b, middle panel). In contrast, the overproduced GFP-tagged zinc finger-mutated Ams2 (MZF-GFP) was bound at lower levels at the histone gene promoters (Fig. 3b, right panel, grey bars). Notably, however, the Ams2-HA from the chromosomal locus showed little association with the histone gene promoters (Fig. 3b, right panel, white bars). The decreased level of promoter binding by Ams2-HA from the chromosomal locus was effectively the same as that in the cells overproducing wild-type Ams2-GFP cells. The decreased binding to the histone gene promoters was not the result of low levels of protein expression of Ams2-HA, given that the levels of Ams2-HA protein were not altered by overproduction of either the GFP-tagged wild-type or zinc finger-mutated Ams2 (Supplementary Fig. S4). These results indicated that histone gene promoter binding by Ams2 from the chromosomal locus is strongly inhibited by overproduction of the zinc finger-mutated protein, thus impairing induction of transcription of the core histone genes.

### Ams2 self-interacts via the N-terminal region

**Figure 4:**
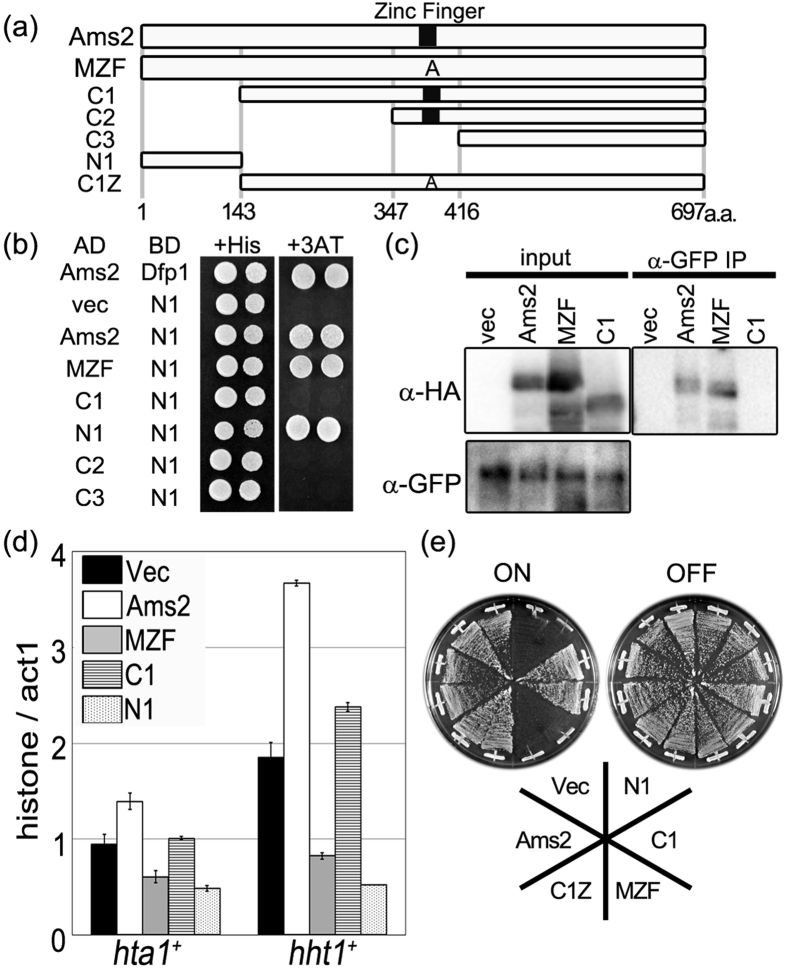
Ams2 self-interacts via its N-terminal region. (**a**) Schematic representation of Ams2 deletion mutants. Amino acid residue numbering is indicated below the panel. (**b**) Various fragments from Ams2 were cloned into the pGAD (AD) plasmid. Each plasmid was transformed into the L40 strain along with either pBTM (BD)-Dfp1 or pBTM-N1. The resulting transformants were grown on medium supplemented with histidine (+His) or lacking histidine and supplemented with 25 mM 3-AT (+3AT). (**c**) Cells encoding GFP-tagged Ams2 (YTP1576) and containing pRep41 (Vec), pRep41-Ams2-HA (Ams2), pRep41-MZF-HA (MZF), or pRep41-C1-HA (C1) were grown in minimal medium in the absence (ON) of thiamine at 33 °C for 18 h and arrested in S phase by exposure to 12 mM HU for 3.5 h. Immunoprecipitation was performed with anti-GFP antibody and precipitated proteins were detected by western blotting with anti-HA antibody. (**d**) Wild-type (HM123) cells containing pRep41 (Vec, black bars), pRep41-Ams2-HA (Ams2, white bars), pRep41-MZF-HA (MZF, grey bars), pRep41-C1-HA (C1, striped bars), or pRep41-N1-HA (N1, dotted bars) were grown in minimal medium in the absence (ON) of thiamine at 33 °C for 18 h. The relative amounts of *hta1*^+^ and *hht1*^+^ mRNA were calculated by normalising to that of *act1*^+^ mRNA. The error bars indicate the standard deviation of two independent cultures. (**e**) *cnp1-1* (Sp525) cells containing pRep41 (Vec), pRep41-Ams2-HA (Ams2), pRep41-MZF-HA (MZF), pRep41-N1-HA (N1), pRep41-C1-HA (C1), or pRep41-C1Z-HA (C1Z) were grown on minimal medium in the absence (ON) or presence (OFF) of thiamine at 26 °C for 5 days.

Histone gene promoter binding by Ams2 is impaired by overproduction of zinc finger-mutated Ams2, raising the possibility that the wild-type Ams2 interacts with the zinc finger-mutated Ams2 protein, and that this interaction prevents histone gene promoter binding by wild-type Ams2. To address this possibility, we used a yeast two-hybrid assay to test whether Ams2 is capable of self-interaction. Having previously shown that Ams2 interacts with Dfp1 (a Dbf4-dependent protein kinase (DDK) subunit)^18^, we used Dfp1 as a positive control. As shown in Fig. 4b and Supplementary Fig. S5, cells expressing BD-N1 and AD-Ams2 were able to grow on 3AT-containing plates (+3AT), indicating physical association between the N-terminal fragment (N1, amino acid residues 1–143) and full-length Ams2. Equivalent experiments demonstrated physical association between N1 and zinc finger-mutated Ams2 (MZF) and among N1 fragments (Fig. 4b). In contrast, the C-terminal fragment C1 (corresponding to amino acid residues 143–697 and notably lacking the N1 region) no longer interacted with N1. This result suggested that Ams2 molecules physically interact with each other, and that this interaction is mediated by the N1 region. Physical interaction of Ams2 via self-interaction was confirmed by co-immunoprecipitation experiments (Fig. 4c). Cells expressing GFP-tagged Ams2 (from the chromosomal locus) were engineered to overproduce (from a plasmid) HA-tagged wild-type Ams2, HA-tagged zinc finger-mutated protein (MZF), or HA-tagged N1 region-deleted protein (C1). The cell lysates were immunoprecipitated with anti-GFP antibody. The overproduced HA-tagged wild-type and zinc finger-mutated Ams2 proteins were co-immunoprecipitated with the Ams2-GFP, but the N1 region-deleted Ams2 (C1) -HA was not. The same results were obtained in three independent experiments. These observations indicated that Ams2 proteins physically interact with each other and that this interaction is mediated by the N-terminal region of the Ams2 protein (amino acid residues 1–143).

The interaction between wild-type Ams2 and the zinc finger-mutated protein prevents histone gene promoter binding and impedes the induction of core histone gene transcription. By extension, these results suggested that overproduction of the N-terminal self-interaction fragment of Ams2 (N1) will also impair histone gene transcription, whereas overproduction of the self-interaction-defective C-terminal fragment of Ams2 (C1) will not. We tested these predictions by using quantitative RT-PCR to determine histone transcription levels in cells that overproduced N- or C- terminal fragments of Ams2. When the N-terminal fragment of Ams2 (N1) was overproduced in wild-type cells, histone gene transcript levels were decreased compared to those seen with empty vector, but were similar to the levels seen in a strain overproducing the zinc finger-mutated protein (Fig. 4d and Supplementary Fig. S6a, dotted bar). Cells that overproduced the C-terminal fragment (C1) did not show a similar decrease in histone gene transcript levels (Fig. 4d, striped bar), but the transcript levels still fell short of the levels seen in a strain that overproduced wild-type Ams2 (described below). A previous study showed that although histone transcript levels are decreased in *Δams2* cells, histone protein levels are not^12^. Similarly, the histone protein levels (H3) were not markedly changed in zinc finger-mutated Ams2-overproducing cells (Supplementary Fig. S6b). These results suggested that the transcriptional activation of wild-type Ams2 protein is impaired by interaction with dysfunctional Ams2 mutant proteins (e.g., zinc finger-mutated or N-terminal fragment Ams2). The zinc finger-mutated Ams2 (MZF) and N-terminal (N1) proteins do not have the functional zinc finger motif; thus, these proteins hardly bind to the histone promoter. These self-interactions are presumably mediated by the N-terminal region N1, given that overproduction of C-terminal fragment C1 (which is deleted for the N1 region) does not impede transcription of the histone genes. In support of this notion, overproduction of the self-interaction domain-deleted zinc finger-mutated Ams2 protein (C1Z, Fig. 4a) did not induce a growth defect in *cnp1-1* cells (Fig. 4e). Taken together, these results indicated that Ams2 proteins interact via the N-terminal region (N1; amino acid residues 1–143); overproduction of non-DNA-binding Ams2 proteins that include the N-terminal region impairs Ams2-mediated induction of histone gene transcription.

### The N-terminal self-interacting domain is required for full activation of histone gene transcription

**Figure 5:**
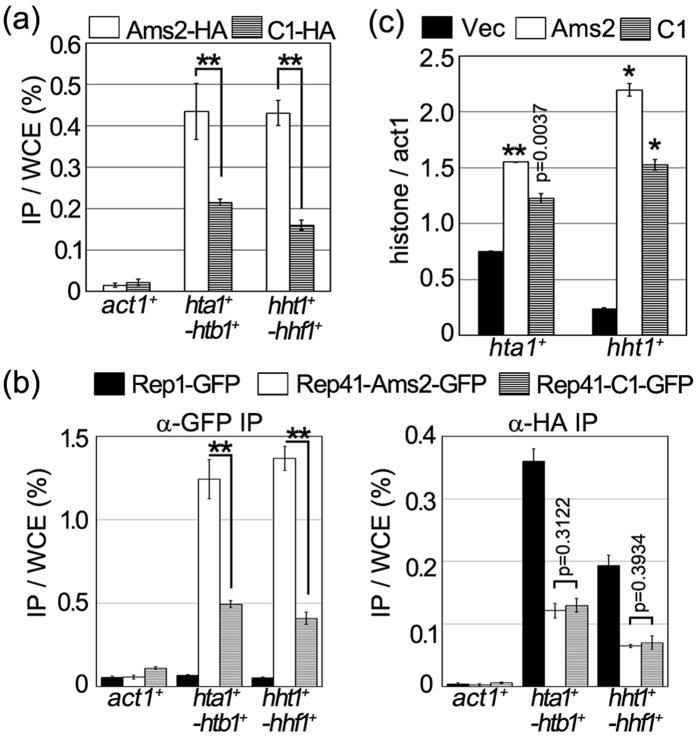
Self-interacting domain of Ams2 is required for full activation of histone transcription. (**a**) Wild-type (HM123) cells containing pRep41-Ams2-HA (white bars) or pRep41-C1-HA (striped bars) were grown in minimal medium in the absence (ON) of thiamine at 33 °C for 22 h. DNAs co-immunoprecipitated with anti-HA antibody were quantified by real-time PCR with promoter region probes specific for *act1*^+^, *hta1*^+^-*htb1*^+^, or *hht1*^+^-*hhf1*^+^. The amount of each immunoprecipitated DNA was divided by that of the corresponding whole-cell extract DNA after background titration. The error bars indicate the standard deviation from two independent cultures. p values were calculated using a Student’s t-test. Double asterisk indicates p < 0.00001. (**b**) Cell lysates were prepared from cells encoding HA-tagged Ams2 (YTP894) and containing pRep41-GFP (black bars), pRep41-Ams2-GFP (white bars), or pRep41-C1-GFP (striped bars) that were grown in minimal medium in the absence (ON) of thiamine at 33 °C for 17 h and arrested in S phase by exposure to 12-mM HU for 3.5 h. DNAs co-immunoprecipitated with anti-GFP (left panel) or anti-HA (right panel) antibodies were quantified by real-time PCR with promoter region probes specific for *act1*^+^, *hta1*^+^-*htb1*^+^, or *hht1*^+^-*hhf1*^+^. The amount of each immunoprecipitated DNA was divided by that of the corresponding whole-cell extract DNA after background titration. The error bars indicate the standard deviation from three independent cell cultures. p values were calculated using a Student’s t-test. Double asterisks indicate p < 0.00001. (**c**) Ams2-deleted (YTP112, *ams2Δ*) cells containing pRep41 (Vec, black bars), pRep41-Ams2-HA (Ams2, white bars), or pRep41-C1-HA (C1, striped bars) were grown in minimal medium in the absence (ON) of thiamine at 33 °C for 18 h. The relative amounts of *hta1*^+^ and *hht1*^+^ mRNA were calculated by normalising to that of *act1*^+^ mRNA. The error bars indicate the standard deviation from two independent transformants and duplicate PCR experiments. p values for comparison with vector control were calculated using a Student’s t-test. Single or double asterisks indicate p < 0.0001 or p < 0.00001, respectively.

As shown above (Fig. 4d and Supplementary Fig. S6a, Ams2 vs. C1), histone gene transcription in cells that overproduced the self-interaction-domain-deleted Ams2 (C1) was not activated to similar levels in cells that overproduced wild-type Ams2. To ascertain whether the low levels of histone gene transcripts reflect histone gene promoter binding by the self-interaction-domain-deleted Ams2 (C1) itself, the level of histone gene promoter binding was examined by ChIP assay. Wild-type cells containing pRep41-Ams2-HA or pRep41-C1-HA plasmids were cultured in the absence of thiamine (derepressed condition). The cell lysates were immunoprecipitated with anti-HA antibody. The accumulation of the overproduced self-interaction-domain-deleted Ams2 (C1) on the histone promoter was decreased compared with that of the overproduced wild-type Ams2-HA (Fig. 5a). Western blot analysis confirmed that the wild-type and C-terminal fragment Ams2 (C1) proteins were produced at similar levels in the respective strains (Supplementary Fig. S7a).

Given that the self-interaction-domain-deleted C1 fragment exhibited decreased accumulation on the core histone gene promoters, we wondered whether cells overproducing the C1 fragment exhibited a compensatory increase in histone promoter binding by Ams2 protein from the chromosomal locus. We accordingly performed a ChIP assay using strains that encoded HA-tagged Ams2 from the chromosomal locus and contained pRep41-Ams2-GFP or pRep41-C1-GFP plasmids. As seen in Fig. 5a, overproduced GFP-tagged C1 protein accumulated on the core histone gene promoters, but the accumulation levels were significantly reduced compared to those seen upon overproduction of GFP-tagged wild-type Ams2 (Fig. 5b, left panel, white and striped bars). We confirmed that the overproduced GFP-tagged wild-type Ams2 or C1 proteins were produced at similar levels, and that the HA-tagged Ams2 protein from the chromosomal locus also was produced at similar levels in these strains (Supplementary Fig. S7b). These results suggested that Ams2-HA accumulation did not change between the self-interaction-domain-deleted C1-GFP- and wild-type Ams2-GFP-overproducing cells (Fig. 5b, right panel). Thus, the self-interaction-domain-deleted Ams2 may be impaired for stable binding to the histone gene promoter, paralleling effects on the levels of histone gene transcripts.

Another possibility is that the self-interaction-domain-deleted Ams2 protein fails to activate histone gene transcription such that histone gene transcription is moderately activated solely by the low levels of binding by the Ams2 protein from the chromosomal locus. To investigate whether the self-interaction-domain-deleted Ams2 (C1) protein activates histone gene transcription, we measured the levels of core histone gene transcripts in an *ams2*^+^ null strain containing the pRep41-C1 plasmid. When the C1 fragment was overproduced in *ams2Δ* cells, the amounts of *hta1*^+^ or *hht1*^+^ gene transcripts were increased compared with those in cells containing a vector control (Fig. 5c and Supplementary Fig. S7c), suggesting that histone gene transcription is activated by the C1 protein. However, the levels of these histone gene transcripts in C1-overproducing *ams2Δ* cells were lower than those in cells that overproduced wild-type Ams2. Thus, while Ams2 lacking the self-interacting domain is capable of activating histone gene transcription, this protein does not induce full activation of histone gene transcription. These results indicated that the moderate activation of histone gene transcription by overexpressed self-interaction-domain-deleted Ams2 can be attributed to low levels of histone promoter binding. The low-level binding by the self-interaction-domain-deleted fragment protein may result in unstable binding to the promoter.

## Discussion

**Figure 6:**
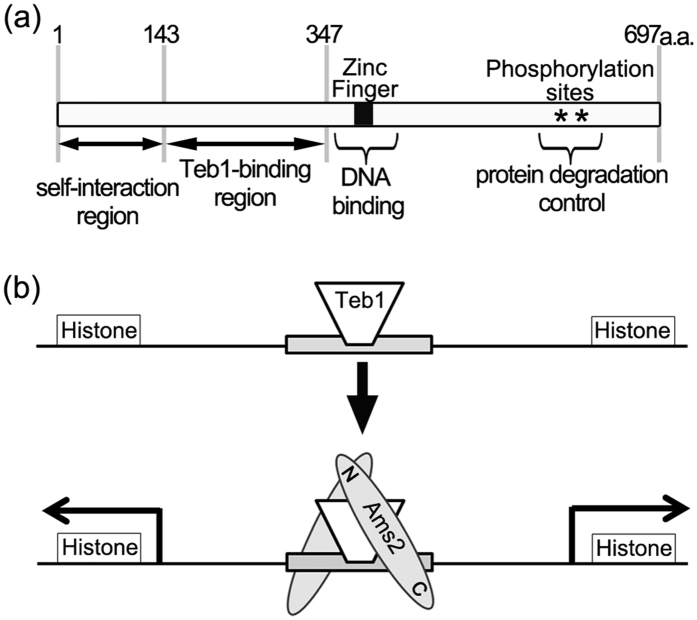
Model for the binding of self-interacting Ams2 to the histone gene promoter region. (**a**) Schematic representation of Ams2 functional domains. Amino acid residue numbers are indicated above the panel. (**b**) Teb1 protein (trapezoid) binds to the TTAGGG sequence in the AACCCT-box (grey box). In S phase, Ams2 (grey ellipses) is produced and Ams2 binds to Teb1 via the Teb1 binding region. At this time, the N-terminus of Ams2 self-interacts (N in grey ellipses) and the zinc finger motif of Ams2 is required for stable binding to histone promoter DNA.

In this study, we characterised the various functional domains of the Ams2 molecule (summarized in Fig. 6a). The N-terminal region of Ams2 (amino acid residues 1–143) is required for self-interaction, and the zinc finger domain functions in DNA binding. We also identified an interaction between the N-terminal region of Teb1 (amino acid residues 1–235) and the middle region of Ams2 (amino acid residues 143–347). The C-terminal region of Ams2 is known to contain phosphorylation sites that are essential for cell cycle-specific Ams2 degradation^18^. We previously showed that Ams2 physically interacts with, and is phosphorylated by, DDK^18^, raising the possibility that DDK may bind near these phosphorylation sites.

Overproduction of the zinc finger-mutated Ams2 induced chromosome missegregation and attenuation of CENP-A localisation. Chen and collaborators^17^ have shown that Ams2 associates with centromere DNA, suggesting that this association promotes the incorporation of CENP-A into the centromere nucleosome. Our results rule out the possibility that the zinc finger-mutated Ams2 is prevented from direct incorporation into the centromere nucleosome. However, we note that fission yeast exhibits two distinct phases of CENP-A loading, corresponding to the S and G2 phases of the cell cycle^22^,^24^. In *ams2Δ* cells, CENP-A fails to be retained during S phase, but re-accumulates on the centromere during G2 phase^22^. This observation implies that S phase centromere localisation of CENP-A depends on the presence of Ams2, while the G2-phase localisation does not^22^. Even if the zinc finger-mutated Ams2 interferes with centromere nucleosome formation, CENP-A might be able to bind to the centromere by Ams2-independent mechanisms. Therefore, the phenotype caused by the zinc finger-mutated Ams2 is most likely attributable to the decrease in histone gene transcription. Another Ams2 binding region, distinct from the core histone gene promoter, and containing a 17-bp AACCCT-box sequence, exists upstream of the *SPAC631.02*^+^ gene^12^. Since we could not detect any association of the zinc finger-mutated Ams2 with this region, the zinc finger domain of Ams2 is important for binding to the AACCCT-box sequence. Although the AACCCT-box does not perfectly match the GATA consensus sequence, two sequences (5′-GATn-3′ and 5′-GtTA-3′) with similarity to the GATA consensus can be discerned in the complementary strand of the AACCCT-box^12^. Given that the GATA-type zinc finger of Ams2 mediates self-interaction, the two GATA-like sequences may be recognised by a pair of self-interacting Ams2 proteins. In budding yeast, the zinc finger protein Spt10 forms homodimers via an N-terminal region, and this homodimerisation is believed to facilitate the stable binding of Spt10 to pairs of UAS elements in the histone locus promoters^7^. By analogy with Spt10, self-interacting Ams2 proteins may be stably bound to the promoters of fission yeast histone genes, such that the stabilised binding leads to increased activation of histone gene transcription. The paired histone genes encoding H2A and H2B or H3 and H4 are arranged divergently, with bidirectional transcription of each pair from a central promoter^9^,^10^. To date, the mechanism of bidirectional histone gene transcription remains unclear. In this context, we note that histone gene expression levels appear to be slightly decreased in cells overexpressing Ams2 C1, which is deleted for the self-interaction domain. We postulate that that this phenotype may result from an imbalance of bidirectional transcriptional activity, reflecting unstable binding to the histone promoter.

Teb1 protein binds to many promoter regions that contain the TTAGGG motif, including the core histone gene promoters. Ams2 binding to histone gene promoters requires the presence of Teb1, leading to the suggestion that Teb1 provides a platform for Ams2 binding^21^. Teb1 exhibits nuclear localisation throughout the cell cycle^21^. In contrast, Ams2 protein accumulates at S phase; therefore, Teb1 might be binding to the TTAGGG sequence in the AACCCT-box before Ams2 binds to the histone promoter (Fig. 6b). Teb1 binding to the histone promoter is used as a marker of Ams2 binding at S phase; however, zinc finger-mutated Ams2 cannot bind to the promoter. Thus, stable binding to the histone promoter is ensured by the presence of the zinc finger motif in Ams2. In budding yeast, expression of the zinc finger protein Spt10 is independent of the cell cycle. In contrast, Spt10’s binding partner, Spt21, is expressed in a cell cycle-dependent fashion, with peaks of expression occurring during S phase^25^. We hypothesise that Ams2 and Teb1 may play roles in fission yeast equivalent to the respective functions of Spt10 and Spt21 in budding yeast.

In contrast to the situation in yeasts, the core histone genes in higher eukaryotes are highly iterated and organised into clusters. In these organisms, the histone gene clusters are associated with the histone locus body (HLB)^26^. The HLB is one of the nuclear bodies that have been reported in *Drosophila* and human cells, and are required for histone gene mRNA biosynthesis^27^. The *Drosophila* HLBs are associated with the S phase-specific histone gene loci. Although it is unknown whether fission yeast histone genes form a nuclear body, Ams2 forms some foci in the nucleus at S phase^17^. Thus, binding of Ams2 to the core histone gene promoters may lead to nuclear body assembly by self-interaction activity. This process may lead to formation of a structure analogous to the nuclear body, in this instance using Ams2 as a scaffold. It remains to be determined whether Ams2 forms dimers or multimers, and whether fission yeast histone genes form a nuclear body.

## Methods

### General techniques, media, and strains

General fission yeast techniques and media have been previously described^12^. The fission yeast strains used in this study are listed in Supplementary Table S1.

### Cell cycle experiments

Cell cycle experiments were performed using wild-type (HM123), *cnp1-1* (Sp525), or *ams2*^+^ null (YTP112) strains, or with an isogenic strain encoding HA-tagged Ams2 (YTP894), or GFP-tagged Ams2 (YTP1576). Cells of these strains (all harbouring *nmt* promoter (pRep41-based) plasmids) were pre-cultured in minimal medium in the presence of 2 μM thiamine. After four washes with distilled water, the cells were cultured in minimal medium in the absence of thiamine at 26 °C (for *cnp1-1*) or 33 °C (for other cells). For S phase synchronisation, the cultured cells grown in the absence of thiamine were arrested by adding 12 mM hydroxyurea (HU). In fission yeast, asynchronous culture cells are mainly in G2 phase. When cells were not treated with HU, Ams2 protein expressed from the chromosomal locus was barely detectable. Therefore, to assay Ams2 protein (expressed from chromosome), the cells were arrested in S phase by HU (Figs 1b,d, 3b, 4c and 5b).

### RNA preparation and quantitative reverse transcription polymerase chain reaction (qRT-PCR)

*S. pombe* total RNA was extracted from 40-ml volumes of cultured cells by the acid–phenol method, as previously described^28^. Aliquots (0.5 μg per strain) of total RNA were used for the synthesis of cDNA by the ReverTra Ace qPCR RT Master Mix with gDNA Remover (TOYOBO) in accordance with the manufacturer’s instructions. The synthesised cDNAs were quantified using a 7500 Fast Real-time PCR system (Life Technologies) and Gene Ace SYBR qPCR Mix α Low Lox (Nippongene). Overall efficiencies of quantitative RT-PCR were calculated as previously described^12^. Expression profiles for individual histone cDNAs were normalised against *act1*^+^ cDNA levels. The primer sequences were as previously reported^12^. Data are presented as mean ± standard deviation based on two independent cultures and duplicate PCR experiments.

### Microscopy

DAPI staining was performed as described^22^. Cnp1^ts^-GFP expression (Sp1102), or *cnp1-1* (Sp525) cells containing pRep41, pRep41-Ams2, or pRep41-MZF plasmid were pre-cultured in minimal medium in the presence of 2 μM thiamine. After four washes with distilled water, the cells were cultured in minimal medium in the absence of thiamine at 26 °C for 30 h. Cells then were fixed in methanol at −80 °C, washed with phosphate-buffered saline (PBS), and mixed with 200 ng/ml 4,6-diamino-2-phenylindole (DAPI). Stained cells were observed with a DM5500B digital microscope equipped with a 100× objective lens (N.A. 1.30) and a DFC 310FX digital CCD colour camera (Leica Microsystems). The frequency of unequal segregation was determined in binucleate cells. Data are presented as mean ± standard deviation based on four independent experiments.

### Chromatin immunoprecipitation (ChIP) assay

ChIP assay was performed as previously described^18^,^22^. The immunoprecipitated DNA was analysed by real-time PCR using a 7500 fast (Life Technologies) and Gene Ace SYBR qPCR Mix α Low Lox (Nippongene). The primer sequences used were as previously reported^12^. Data are presented as mean ± standard deviation based on two independent cultures and duplicate PCR experiments.

### Western blotting analysis

Protein extracts for western blotting were prepared from trichloroacetic acid (TCA) -treated cells. For ChIP samples, aliquots of the formaldehyde-fixed cells were used. Briefly, cells were collected and washed in 500 μl of ice-cold distilled water. Then, 150 μl of ice-cold lysis buffer (1.85 M NaOH, 7.5% β-mercaptoethanol) was added, and the suspension was held on ice for 10 min. TCA was then added to a final concentration of 25%. The suspension was held on ice for 10 min. Proteins were pelleted by centrifugation at 18,000 × *g* for 10 min at 4 °C and then suspended in 1× gel-loading sample buffer. The protein samples were separated by SDS-polyacrylamide gel electrophoresis, blotted onto a membrane, and analysed by western blotting using anti-HA (12CA5, Roche, 11583816001), anti-GFP (Roche, 1814460), anti-H3 (Abcam, ab1791), and anti-PSTAIR (Sigma, P7962) antibodies.

### Yeast two-hybrid assay

Plasmids were transformed into budding yeast strain L40, the genotype of which is *MATa his3Δ200 leu2-3,112 trp1-901 ade2 LYS2::(LexAop)*_*4*_*-HIS3 URA3::(LexAop)*_*8*_*-lacZ*, and the transformants were grown on medium lacking tryptophan and leucine (+His), or on medium lacking tryptophan, leucine, and histidine but supplemented with 25 mM 3-amino-1,2,4,-triazole (+3AT) at 30 °C.

### Co-immunoprecipitation

Cell lysates were prepared in IP buffer (50 mM HEPES pH 7.5, 250 mM NaCl, 5 mM MgCl_2_, 1 mM DTT, 0.1% NP40, 5% glycerol, 5 mM NaF, 1 mM Na_3_VO_4_, 0.05 mM spermine, 0.125 mM spermidine, 1 mM PMSF, 1x cOmplete™ Protease Inhibitor) and broken using glass bead-beating with a FastPrep24 (MP Biomedicals). Aliquots of the clarified lysates, each containing 5 mg total protein, were incubated with anti-GFP antibody (Roche, 1814460) for 2 h at 4 °C followed by addition of Dynabeads anti-Mouse IgG (Veritas) for 3 h at 4 °C. The beads were washed 4 times with IP buffer and then suspended in 1x sample buffer and boiled.

## Additional Information

**How to cite this article**: Takayama, Y. *et al*. Characterisation of functional domains in fission yeast Ams2 that are required for core histone gene transcription. *Sci. Rep.* **6**, 38111; doi: 10.1038/srep38111 (2016).

**Publisher's note:** Springer Nature remains neutral with regard to jurisdictional claims in published maps and institutional affiliations.

## Supplementary Material

Supplementary Information
