# Supplementary material for: Characterisation of functional domains in fission yeast Ams2 that are required for core histone gene transcription
Source: Sci Rep. 2016 Nov 30;6:38111. doi: 10.1038/srep38111 (PMC5128866; doi:10.1038/srep38111)
Supplement: Supplementary Information [file srep38111-s1.pdf]

Supplementary Information

Characterisation of functional domains in fission yeast Ams2 that are required for core histone gene transcription

Yuko Takayama[1,2,*], Masaki Shirai[2] and Fumie Masuda[3]

[1] Department of Biosciences, School of Science and Engineering, Teikyo University, Utsunomiya, Tochigi, 320-8551, Japan

[2] Division of Integrated Science and Engineering, Teikyo University Graduate School of Science and Engineering, Utsunomiya, Tochigi, 320-8551, Japan

[3] Division of Cell Biology, Institute of Life Science, Kurume University, Kurume, Fukuoka, 839-0864, Japan

*To whom correspondence should be addressed.

1-1, Toyosatodai, Utsunomiya, Tochigi, 320-8551, Japan

Tel: +81-28-627-7242; Fax: +81-28-627-7187; Email: takayama@nasu.bio.teikyo-u.ac.jp

Supplementary Table

Table S1. The fission yeast strains used in this study

Strain Name	Genotype	Reference
HM123	*h⁻ leu1-32*	[1]
Sp525	*h⁻ leu1-32 ura4-D18 Δcnp1::ura4⁺ lys1⁺-cnp1-1*	[2]
Sp1102	*h⁻ leu1-32 ura4-D18 lys1⁺-cnp1-1-GFP*	[3]
YTP112	*h⁻ leu1-32 ura4-D18 Δams2::kan*	[4]
YTP894	*h⁻ leu1-32 ams2⁺-3HA-kan*	[4]
YTP1387	*h⁻ leu1-32 ams2MZF-3HA-NAT*	This study
YTP1511	*h⁺ leu1-32 ura4-D18 lys1Δams2::kan*	This study
YTP1543	*h⁺ leu1-32 ura4-D18 lys1 ams2⁺-3HA-kan*	This study
YTP1576	*h⁻ leu1-32 ura4-D18 ams2⁺-GFP-ura4⁺*	This study

Supplementary Figures

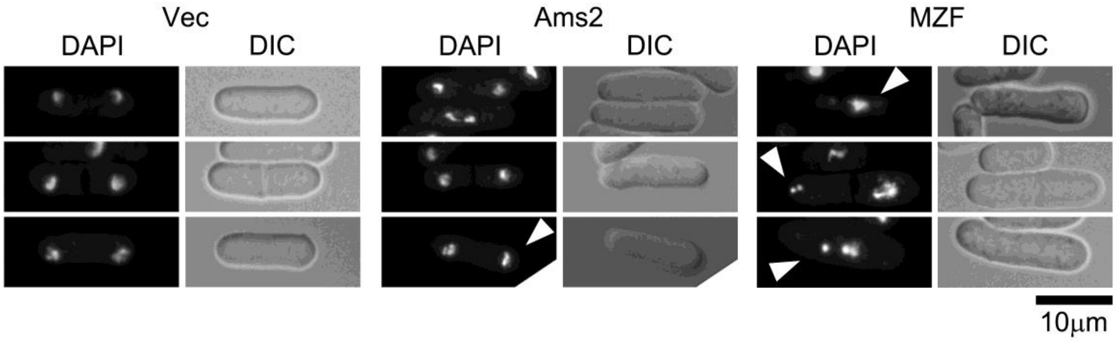

Figure S1. Overproduction of zinc finger-mutated Ams2 in *cnp1-1* causes chromosome missegregation. *cnp1-1* (Sp525) cells containing pRep41 (vec), pRep41-Ams2 (Ams2), or pRep41-zinc finger mutant (MZF) were grown in minimal medium in the absence of thiamine at 26°C for 36h. Cells were fixed with methanol and stained with DAPI. Arrowheads indicate the chromosome missegregation.

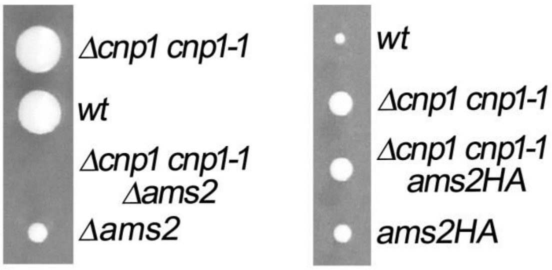

Figure S2. Double mutant *cnp1-1 ams2Δ* cells exhibit a synthetic growth defect. *cnp1-1* (Sp525) was crossed with *ams2Δ* (YTP1511) or *ams2-HA* (YTP1543) and sporulated. The spores were dissected and incubated on YES plates at 25°C.

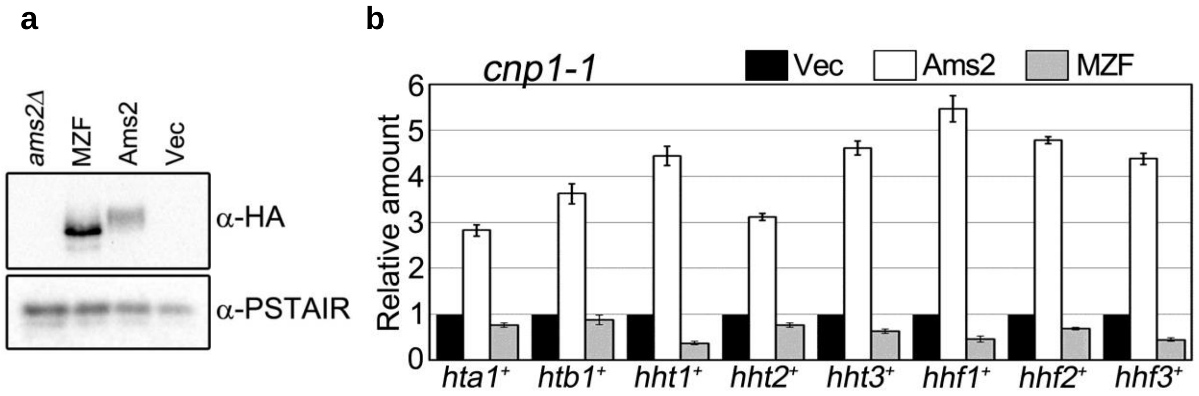

Figure S3. Overproduced zinc finger-mutated Ams2 protein down-regulates core histone gene transcription. (a) Wild-type (HM123) cells containing pRep41 (vec), pRep41-Ams2-HA (Ams2), or pRep41-zinc finger-mutated Ams2-HA (MZF) were grown in minimal medium in the absence of thiamine at 33°C for 22 h. Lysates prepared from the cells were processed and subjected to western blotting with anti-HA (to detect the overproduced plasmid-encoded protein), or anti-PSTAIR (loading control). (b) *cnp1-1* (Sp525) cells containing pRep41 (Vec, black bars), pRep41-Ams2 (Ams2, white bars), or pRep41-zinc finger mutant (MZF, grey bars) were grown in minimal medium in the absence of thiamine at 26°C for 30 h. The transcript levels for the indicated genes were determined by quantitative RT-PCR. The transcript levels were calculated from two independent experiments. The relative amounts of each mRNA were divided by that of the corresponding vector control after normalizing to that of *act1*$^+$ mRNA. Values are presented as mean ± standard deviation.

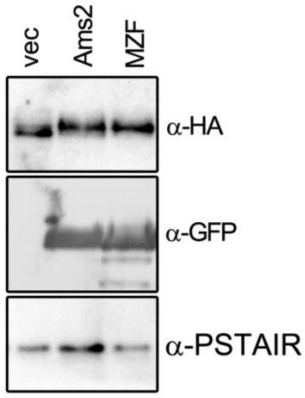

Figure S4. Wild-type or zinc finger-mutated Ams2 protein levels as assessed by western blotting. Cells encoding HA-tagged Ams2 (YTP894) and containing pRep41-GFP (vec), pRep41-Ams2-GFP (Ams2), or pRep41-zinc finger-mutated Ams2-GFP (MZF) were grown in minimal medium in the absence of thiamine at 33°C for 18 h and arrested in S phase by incubation with 12 mM hydroxyurea for 3.5 h. Lysates prepared from the cells were processed and subjected to western blotting with anti-HA (to detect the Ams2 protein expressed from the chromosomal locus), anti-GFP (to detect the overproduced plasmid-encoded protein), or anti-PSTAIR (loading control).

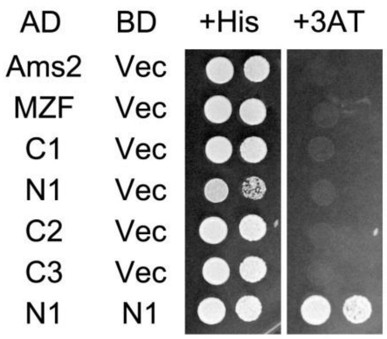

Figure S5. Interaction between Ams2 and Ams2 deletion mutant proteins as assessed by yeast two-hybrid assay. pGAD-derived plasmids were constructed to encode Ams2 as the wild-type (Ams2) or mutant (MZF, C1, C2, C3, N1) protein fused to the Gal4 activation domain (AD); separate pBTM116-derived constructs were designed to encode the LexA binding domain (BD) alone or fused to the N1 fragment of Ams2. These plasmids were transformed into L40 yeast cells. Independent transformants encoding the indicated constructs were spotted to medium supplemented with histidine (+His) or lacking histidine but supplemented with 25 mM 3-AT (+3AT). These plates were incubated at 30°C.

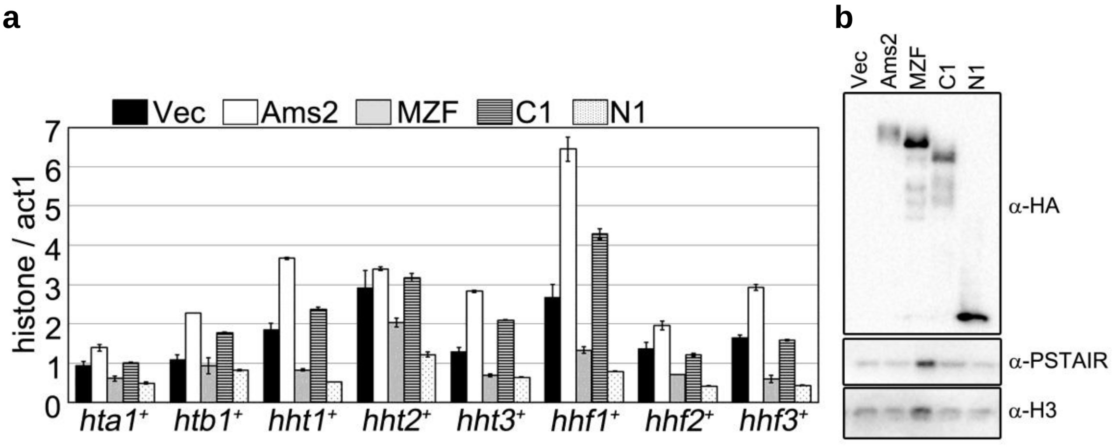

Figure S6. N-terminal region of Ams2 is required for the full activation of histone gene transcription. (a) Wild-type (HM123) cells containing pRep41 (Vec, black bars), pRep41-Ams2-HA (Ams2, white bars), pRep41-MZF-HA (MZF, grey bars), pRep41-C1-HA (C1, striped bars), or pRep41-N1-HA (N1, dotted bars) were grown in minimal medium in the absence of thiamine at 33°C for 18 h. Transcript levels for the indicated genes were determined by quantitative RT-PCR. The relative amounts of core histone gene mRNAs were calculated by normalizing to levels of *act1*$^{+}$ mRNA. Values are presented as mean ± standard deviation of two independent experiments. (b) Lysates prepared from the cells using RT-PCR were subjected to western blotting with anti-HA (to detect the overproduced plasmid-encoded protein), anti-H3, or anti-PSTAIR (as loading control).

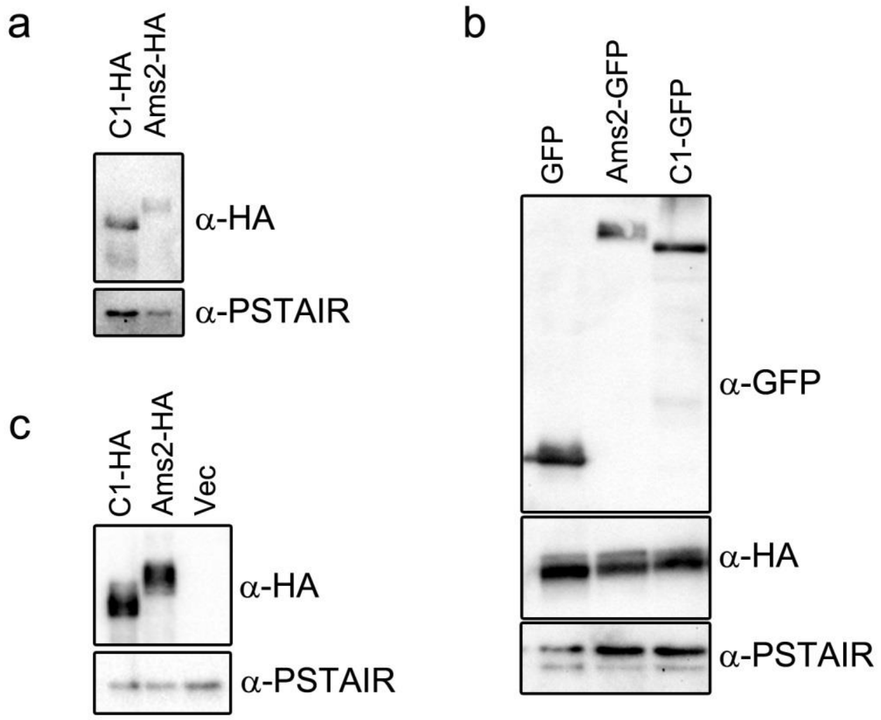

Figure S7. Wild-type or deleted Ams2 protein levels as assessed by western blotting. (a, c) HM123 cells containing pRep41, pRep41-Ams2-HA, or pRep41-C1-HA were grown in minimal medium in the absence (ON) of thiamine at 33°C for 22h (a) or 17 h and arrested in 12 mM HU for 3.5h (c). Cell lysates were subjected to western blotting with anti-HA (to detect the overproduced plasmid-encoded protein) or anti-PSTAIR (loading control). (b) Cells encoding HA-tagged Ams2 (strain YTP894) and harbouring pRep41-GFP (GFP), pRep41-Ams2-GFP (Ams2-GFP), or pRep41-C1-GFP (C1-GFP) were grown in minimal medium in the absence of thiamine at 33°C for 18 h and arrested in S phase by incubation with 12 mM hydroxyurea for 3.5 h. Lysates prepared from the cells were processed and subjected to western blotting with anti-HA (to detect Ams2 protein from the native locus), anti-GFP (to detect the overproduced plasmid-encoded protein), or anti-PSTAIR (loading control).

References

1 Takayama, Y. & Takahashi, K. Differential regulation of repeated histone genes during the fission yeast cell cycle. *Nucleic Acids Res.* **35**, 3223-3237 (2007).

2 Takahashi, K., Chen, E. S. & Yanagida, M. Requirement of Mis6 centromere connector for localizing a CENP-A-like protein in fission yeast. *Science* **288**, 2215-2219 (2000).

3 Takayama, Y. *et al.* Biphasic Incorporation of Centromeric Histone CENP-A in Fission Yeast. *Mol. Biol. Cell* **19**, 682-690 (2008).

4 Takayama, Y. *et al.* Hsk1- and SCF(Pof3)-dependent proteolysis of S. pombe Ams2 ensures histone homeostasis and centromere function. *Dev. Cell* **18**, 385-396, doi:10.1016/j.devcel.2009.12.024 (2010).
